# Physicochemical and Antibacterial Properties of Alginate Films Containing Tansy (*Tanacetum vulgare* L.) Essential Oil

**DOI:** 10.3390/polym15020260

**Published:** 2023-01-04

**Authors:** Jolanta Kowalonek, Natalia Stachowiak, Kinga Bolczak, Agnieszka Richert

**Affiliations:** 1 Faculty of Chemistry, Nicolaus Copernicus University in Torun, Gagarina St. 7, 87-100 Torun, Poland; 2 Department of Genetics, Faculty of Biological and Veterinary Sciences, Nicolaus Copernicus University in Torun, Lwowska St. 1, 87-100 Torun, Poland

**Keywords:** sodium alginate film, tansy (*Tanacetum vulgare* L.) essential oil, antioxidant activity, antibacterial properties

## Abstract

Tansy (*Tanacetum vulgare*) is a common plant used in folk medicine for digestive problems, fevers, and migraines; against parasites; and as an insect repellent. The active substances in essential oil are responsible for its antimicrobial and antioxidant activity. Thus, tansy essential oil (TO) was added to alginate films to fabricate materials with antioxidant and antibacterial properties for food packaging. Sodium alginate films with glycerol and TO were tested in terms of structure, mechanical, thermal, antioxidant, and antibacterial properties. The structure of the films was examined using SEM and an ATR-FTIR spectrophotometer. The addition of TO to the alginate film significantly changed the films’ microstructure, making them rougher and porous. A low-intensity band at 1739 cm^−1^, indicative of the presence of TO, appeared in all spectra of alginate films with TO. Moreover, the studies revealed that essential oil acted as a plasticizer, slightly reducing tensile strength from about 7 MPa to 5 MPa and increasing elongation at break from 52% to 56% for the sample with 2% TO. The alginate films enriched in TO exhibited antioxidant properties (280 μmol Trolox/100 g of the sample with 2% TO) and antibacterial activity against *Escherichia coli*, *Staphylococcus aureus*, and *Pseudomonas aeruginosa*.

## 1. Introduction

Implementing biodegradable and renewable natural biopolymers can reduce the harmful effects and environmental pollution caused by non-biodegradable plastics [1,2]. Biodegradable polymers can be an alternative to achieving the sustainability of the plastics industry and serve as a possible solution for overcrowded landfills [3]. The considerable amount of nondegradable polymers used in packaging has led to the emergence of biodegradable plastics, mainly for food packaging and bioplastic industries [4]. Synthetic polymers applied in food packaging are gradually being replaced by biopolymers, such as polysaccharides (starches [5], cellulose [6], pectins [7], chitosan [8], sodium alginate [9]), proteins (casein [10], gelatin [11], collagen [12]), and polymers obtained by microbial production, e.g., polyhydroxyalkanoates (PHA) [13].

Among these biopolymers, sodium alginate is readily available and has widespread applications. Sodium alginate, mainly extracted from the cell walls and intercellular spaces of marine brown algae, is a linear chain polysaccharide that contains two structural units of 1–4 linked α-L-guluronic acid and β-D-mannuronic acid [14,15]. This natural polymer is characterized by film-forming properties, resistance to oxygen permeability, and biocompatibility, making it an excellent component for producing functional packaging materials and in the medical field [16,17,18,19]. Alginates are used as an emulsifier, stabilizer, thickening agent, and flavor adjuvant. This biopolymer also exhibits properties such as a high capacity to incorporate and release substances, cell affinity, and strong bioadhesion. As a result, it is an ideal polymer for producing hydrogels, scaffolds, tablets, nanoparticles, liposomes, and microparticles [20]. Nevertheless, alginates display poor mechanical strength and loss of structural integrity, which limits their applications [20].

The film-forming ability of alginate was used to prepare films functionalized by adding essential oils with various properties. Essential oils are natural aromatic volatile liquids extracted from, i.e., flowers, buds, leaves, bark, roots, fruits, peels, seeds, and resin of plants [21,22]. They are secondary metabolites of plants. There are several extraction methods, but steam distillation is the most popular technique for producing essential oils [23].

Among various essential oils, tansy (*Tanacetum vulgare*) essential oil (TO) is extracted from flower baskets or leaves of tansy, and its main components are 1,8-cineole, β -thujone, α-thujone, *cis*-chrysanthenol, borneol, myrtenol, camphor, *trans*-chrysanthenyl acetate, artemisia ketone, (E)-dihydroxycarvone, *trans*-chrysanthenol, bornyl acetate, camphene, sabinene, and carvone [24,25,26,27,28,29,30,31,32]. Tansy is a perennial plant belonging to the Asteraceae family. It grows natively in Europe and Asia in a moderate climate and in North America as introduced species that quickly adapts to new environments [25,26,28,31]. It grows wildly along roadsides, balks, banks of rivers, and wastelands [26,31], achieving 150 cm of height and forming yellow button-like flowers [28,33]. Its stems are usually branched from the bottom to the top, and its leaves are similar to fern leaves [28]. The intraspecific diversity of this plant is enormous. The composition of tansy essential oil varies considerably depending on its geographic origins. Simultaneously, the chemotypes of this oil have been identified according to the first dominant constituent [28]. A compound of at least 40% determines the chemotype of the plant [34]. Tansy essential oil has interesting biological features, including anthelmintic and antibacterial properties [26,35]. Moreover, it has important anti-inflammatory, diuretic, and antioxidant properties [36]. The advantages of tansy for processing bioactive components in foods and medicinal or agricultural usage have been revealed. Tansy products (essential oils and extracts) have been applied in cosmetics, perfumery, photography, and culinary [33,37]. As an herbal, tansy has been traditionally used in lotions, dyes, repellents against insects, preservatives, and as a remedy for migraine, neuralgia, rheumatism, and loss of appetite [34].

To the best of our knowledge, polymer materials loaded with tansy (*Tanacetum vulgare*) essential oil intended for food packaging have not yet been investigated. This research aimed to prepare sodium alginate-based films with the introduced tansy essential oil and to test their physicochemical, antimicrobial, and antioxidant properties. These films were proposed to be used as food packaging; thus, they should protect food from oxidation and human pathogens to extend its shelf life. Three common human pathogens, *Escherichia coli*, *Staphylococcus aureus*, and *Pseudomonas aeruginosa*, were chosen for the tests.

## 2. Materials and Methods

### 2.1. Materials

Sodium alginate (ALG) was acquired from Büchi Labortechnik AG (Flawil, Switzerland), glycerol (G) and methanol (pure for analysis) were bought from Avantor Performance Materials Poland S.A. (Gliwice, Poland), tansy essential oil (TO) from *Tanacetum vulgare* was purchased from Herbapol w Krakowie SA (Cracow, Poland), a surfactant TWEEN 80 was bought from Greenaction (Kielce, Poland), and 2,2-diphenyl-1-picrylhydrazyl (DPPH, 95%) and 6-hydroxy-2,5,7,8-tetramethylchromane-2-carboxylic acid (Trolox, 97%) were supplied by Sigma–Aldrich (Poznań, Poland).

### 2.2. Determination of Sodium Alginate Molecular Weight

Sodium alginate molecular weight was determined using the viscometric method. The biopolymer was dissolved in 0.1 M NaCl aqueous solution and placed in a Ubbelohde viscometer (type 532 10, K constant 0.01 mm^2^/s) immersed in water bath CT72/P (Si Analytics, Mainz, Germany) at 25 °C. The flow time of sodium alginate solutions of different concentrations was measured automatically with a Viscoclock Plus (Si Analytics, Mainz, Germany) device.

The limiting viscosity number, [η], was estimated from Huggins and Kraemer equations equaling 993 [cm^3^/g]. Next, the limiting viscosity number was taken to calculate the viscosity-average molecular weight based on the Mark-Houwink-Sakurada equation, $\left[\eta \right]=K\bar{M_{v}^{a}\;}$. The viscosity-average molecular weight was 55,800 for K = 0.0178 cm^3^/g and a = 1 [38].

### 2.3. Preparation of Solutions and Films

Sodium alginate was dissolved in distilled water to form a 2% (*w*/*v*) aqueous alginate solution. To this solution, glycerol in the amount of 2.5% (*w*/*v*) was added. Separately, tansy essential oil was mixed with the surfactant with a volume ratio of 2:1. Subsequently, various amounts of this mixture were added to 30 cm^3^ portions of sodium alginate solution with glycerol. These solution portions were poured into Petri dishes to evaporate the solvent. 1%; 1.33%; 1.67%; or 2% (*w*/*v*) of tansy essential oil was added to the biopolymer solution. After water evaporation, the biopolymeric films enriched in tansy essential oil were obtained, and the content of the essential oil in these films with respect to the weight of the biopolymer was as follows: 50%, 66%, 83%, and 100%, respectively.

The film thickness was measured with a digital gauge (Sylvac GC-050, Yverdon, Switzerland) with an accuracy and resolution of 0.001 mm. The thickness values are the averages of several measurements for each sample.

### 2.4. Viscosity Measurements

The viscosity studies were conducted using a DV1 viscometer (Brookfield Ametek, Middleboro, MA, USA) viscometer equipped with spindle no. 5. The tests were conducted in solutions; therefore, 2% aqueous solutions of sodium alginate with glycerol and tansy essential oil of different concentrations were prepared in 100 mL beakers, to which the spindle was immersed in the determined position. The rotation speed was in the range of 1.5 to 60 rpm. The temperature of the environment where the measurements were conducted was 20 °C. The tests were performed three times for each solution.

### 2.5. ATR-FTIR Spectroscopy

Infrared spectra of the samples were collected with a Nicolet iS5 (Thermo Fisher Scientific, Waltham, MA, USA) spectrophotometer with ID7 ATR equipment containing ZnSe crystal, whose angle of incidence was 45°. The apparatus operating parameters were applied: 4 cm^−1^ resolution, 32 scans, and the range of wavenumbers 4000–550 cm^−1^.

### 2.6. The Uniaxial Tensile Tests

Tensile tests were conducted with a mechanical testing machine EZ-Test SX Texture Analyzer (Shimadzu, Kyoto, Japan). Paddle-shaped samples were cut out of the films, and then these samples were stretched until rupture with a stretching speed of 10 mm/min. The device registered a stress-strain curve from which quantities such as Young’s modulus (E), stress (σ) and strain (ε) at break were calculated. Five tests for each film type were conducted, and the average values were calculated. Trapezium X software version 1.4.5 (Shimadzu, Kyoto, Japan) was used to calculate the mentioned quantities.

### 2.7. Moisture Content

Moisture content (Mc, %) in the films was tested gravimetrically. The samples were dried to a constant weight in an oven at 105 °C. The obtained values were the averages of three repetitions. The following Formula (1) was used for the calculation of moisture content:

(1) $$M_{c},\%=\frac{W_{0}-W_{d}}{W_{0}}100\%,$$

where W_0_ is the sample weight before drying, and W_d_ the sample weight after drying.

### 2.8. UV-VIS Spectroscopy

The UV-VIS spectra of the solutions were performed using a UV-1601 PC spectrophotometer (Shimadzu, Kyoto, Japan).

### 2.9. Scanning Electron Microscopy

The images of the studied samples were performed using a scanning electron microscope (SEM), a model 1430 VP, 2001 (LEO Electron Microscopy Ltd., Cambridge, UK), with an acceleration voltage of 10 kV. Before observation, the samples were covered with about a 10 nm layer of gold and palladium. Images of the surfaces and the fractures of the samples were obtained. To get the fracture, a sample was immersed in liquid nitrogen and then mechanically broken.

### 2.10. Thermal Analysis

The thermal stability of the prepared samples was tested on a thermoanalyzer SDT 2960 Simultaneous DSC/TGA analyzer (TA Instruments, New Castle, DE, USA) in a nitrogen atmosphere with a heating rate of 10 °C/min to 600 °C. Thermogravimetric (TG) and derivative thermogravimetric (DTG) curves were used to determine the characteristic parameters such as T_onset_ (°C), the temperature of the beginning of the process; T_max_ (°C), the temperature at which rate of the process was the maximum (maximum on DTG curve); Δm (%), the weight loss during the process; and a residue (%) at 600 °C after the degradation of the sample.

### 2.11. Antioxidant Capacity

The antioxidant capacity (AC) of the films was tested using DPPH (2,2-diphenyl-1-picrylhydrazyl) radical and applying the QUENCHER method [39], which is based on the direct contact of a solid film with DPPH solution. The tested film was insoluble in the radical solution, and the reactions occurred at the sample’s surface. To determine the AC of the prepared films, 0.1 g of the film was ground in a mill and placed in a test tube to which 6 cm^3^ of 60.86 µmol/dm^3^ DPPH solution in methanol was added. Then, the test tube was put to a shaker (Thermoshaker, VWR International, LLC, Gdańsk, Poland) at 650 rpm for 15 min. Afterward, the test tube was kept in the dark place for 15 min, and then the UV-VIS spectrum of supernatant was registered at 517 nm using a UV-VIS spectrophotometer. The analysis was conducted in triplicate. The percentage of scavenging of the DPPH radical was calculated according to Formula (2):

(2) $$\%\mathrm{DPPH}=\frac{A_{0}-A_{s}}{A_{0}}100\%,$$

where A_0_ is the absorbance of DPPH solution, and A_s_ the absorbance of DPPH solution after contacting the studied film.

A calibration curve based on Trolox solutions showing a linear dependence of the percentage of DPPH scavenging (% DPPH) on Trolox concentration was prepared. The AC of the tested film was expressed in μmol of Trolox equivalents per 100 g of the studied film [40].

### 2.12. Antibacterial Assay

The prepared films were examined in terms of antibacterial activity, which was carried out based on the standard ISO 20645: 2006 [41]. A disk diffusion method was applied for this assessment. The following bacteria strains were used in these tests: *Escherichia coli* (ATCC 8739), *Staphylococcus aureus* (ATCC 6538P), and *Pseudomonas aeruginosa* (ATCC 13388) (Microbiologics^®^, St. Cloud, MN, USA). The agar medium was inoculated with the bacterial culture at a concentration of 1.5 × 10^8^ CFU/mL (0.5 McFarland). The agar medium (AM, Oxoid Company, Napean, ON, Canada) containing the components [g/L] tryptone peptone—15, phyton peptone—5, sodium chloride—5, and agar-agar—15 was poured onto Petri dishes to form a gel. The tested and the control sample had a circle shape with a 25 ± 5 mm diameter, and they were put in the inoculated agar medium. The samples studied were incubated at 37 ± 1 °C for 20 h. After the end of the incubation time, the films were taken off and the presence or absence of zones inhibiting the growth of microorganisms was determined by ISO standard [41]. The analyses were repeated four times.

To check the antibacterial properties of the films quantitatively, the analyses were performed using the ISO 22196:2011 standard [42].

Specified amounts of bacterial cells (10^6^) were placed on sodium alginate film—a control sample—and the biopolymeric films with tansy essential oil. After 0 h for the reference sample and 24 h for the reference and tested samples, the bacteria were retrieved from the films’ surfaces and placed in a neutralizing solution. The number of cultured cells was then determined by placing them in a plate count agar (PCA; Oxoid Company, Nepean, ON, Canada) medium, which is used to determine the total bacterial growth of a sample. The incubation of microorganisms on plates containing the medium was carried out at 35 °C for 48 h. Antibacterial activity (R) was determined according to the standard [42].

### 2.13. Statistical Analysis

One-way ANOVA with Tukey’s post hoc analysis (*p* < 0.05) was performed to compare the results statistically. Different letters (a–e) within the same column indicate significant differences between the compared values.

## 3. Results and Discussion

### 3.1. Viscosity Measurement Results

The dynamic viscosities of aqueous alginate solutions containing glycerol and tansy essential oil are presented in Table 1. Generally, for all solutions, one could notice the same trend of changes in the solutions’ viscosities against speed. At low speeds, the viscosity of the solutions increased, achieving a maximum of 12 rpm, and then decreased. Such behavior suggests shear thickening at lower speeds and shear thinning at higher speeds. At low speeds, the macromolecular chains of alginate can be extended and entangled, resulting in an increase in the solution viscosity. In contrast, at higher speeds, biopolymer chains can be deformed and oriented in the direction of flow, leading to a decrease in the viscosity of the solution. Becker et al. [43], Blaeser et al. [44], and Dodero et al. [45] observed the shear-thinning effect at higher shear rates for a sodium alginate solution. This behavior of the alginate solution persisted ever after hydrophobic tansy essential oil was added. TO with a surfactant was added to the alginate solution to facilitate the mixing of the components. The emulsions formed exhibited significantly higher viscosities than the alginate solution’s viscosity, which was more distinct for the higher rotation speeds applied. The presence of essential oil droplets increased the liquid resistance exerted on the spindle. However, the alginate solution with the highest TO content revealed lower viscosity than the other solutions with this oil, suggesting changes in the sizes of oil droplets in this solution. Bonilla et al. [46] and Salvia-Trujillo et al. [47] measured the viscosity of biopolymer solutions with essential oils. They observed different behaviors of the studied solutions due to the type of essential oil and droplet size. They claimed that the solutions containing smaller droplets of essential oil had lower viscosities than solutions with essential oil forming larger droplets. This may explain the lower values of the viscosities of the alginate solution with the highest TO content compared to those with lower TO content.

### 3.2. ATR-FTIR Spectroscopy Results

The infrared spectra of the samples studied were registered to check how tansy essential oil affected the ordering of the alginate film. The observed absorption bands in the plasticized alginate film were analyzed, and the assignment of suitable bond vibrations is listed in Table 2.

**Figure 1:**
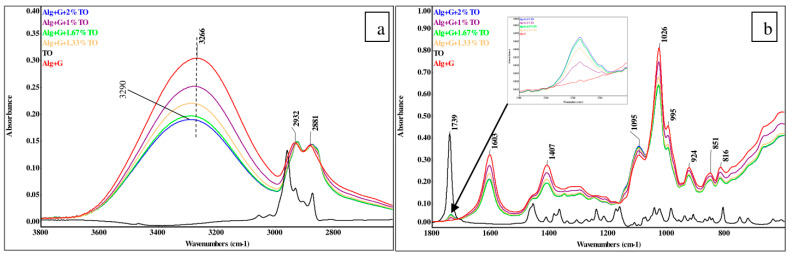
ATR-FTIR spectra of the plasticized alginate film (Alg + G), alginate films with tansy essential oil (Alg + G + TO), and tansy essential oil (TO). (**a**) the spectra in the range of 2600–3800 cm^−^^1^, (**b**) the same spectra in the range of 600–1800 cm^−^^1^.

Figure 1 presents infrared spectra of the sodium alginate film plasticized with glycerol, the plasticized alginate films containing various amounts of TO, and the TO itself. As seen in Figure 1, the spectra of the alginate films enriched in TO were similar to the alginate film’s spectrum without this additive. However, there are differences related to the band intensities, band shifts, and appearance of a new absorption band. That is, almost all absorption band intensities decreased with increasing TO content in the films, which could be related to the lower content of water molecules in the films when hydrophobic substances were added. Moreover, the observed slight shifts of absorption bands both towards the higher and lower wavenumbers indicated weak interactions between the film components. A clear band shift towards higher wavenumbers was noticed for the band of the stretching vibrations of O–H bonds registered at 3266 cm^−1^ in the alginate spectrum but at 3290 cm^−1^ in the sample spectrum with the highest additive content, indicating the reduction in the content of hydrogen bonds. Furthermore, in the spectra of the plasticized alginate films enriched in TO, a new band at 1739 cm^−1^ appeared, and it came from the C=O vibrations of the compounds present in this oil. The intensity of this band increased with increasing essential oil content in the samples (Figure 1b, insert).

Analyzing the spectrum of pure tansy essential oil, the most intense band of C=O bond vibration was detected at 1739 cm^−1^, which indicated the presence of compounds with carbonyl groups such as thujones, camphor, *trans*-chrysanthenyl acetate, or bornyl acetate [24,25,26,27,28,29,30,31,32]. Moreover, the bands in the ether region 1000–1300 cm^−1^ were also observed, indicating the occurrence of substances with ether linkages, such as 1,8-cineole [24,27,30,31,32]. From the TO spectrum, one can infer that compounds with carbonyl groups are the main components of this oil. No bands in the region of vibrations of the O–H bond and double bonds were detected; however, the compounds with those groups might be present in TO, but their content could not be detected by this method. Thus, the infrared technique provided information that compounds with carbonyl groups dominated in this oil. However, it is known that essential oils contain a vast amount of various compounds.

### 3.3. Mechanical Properties Results

Food packaging is expected to be characterized by good mechanical features, i.e., suitable tensile strength and flexibility to endure the packing and storage process. Mechanical properties, such as stress at break, strain at break, and modulus of elasticity of the tested films, were assessed. The values obtained from uniaxial stretching of the samples are shown in Table 3. The plasticized sodium alginate film possessed the highest values of tensile strength and Young’s modulus but the lowest value of elongation at break, except for the sample with 1.33% TO. Moreover, the σ and E values decreased with increasing TO content in the biopolymeric films. In contrast, ε values generally increased with this additive’s increasing content in the film. The results showed that the alginate films with TO could be stretched to a higher extent using lower forces than the alginate film without TO. Thus, tansy essential oil acted as a plasticizer, making the polymer film more flexible but slightly less resistant to rupture. The microdroplets of essential oil filled the spaces between macromolecules, weakening the polymer chain interactions and increasing distances between alginate macromolecules, facilitating the macrochains sliding [50,51,52,53].

### 3.4. The Film Thickness and Moisture Content Results

Table 4 presents the results of the film thickness measurements. The average thickness of the plasticized sodium alginate film was 0.108 mm. When TO was added to the film, this parameter increased; however, the films with TO were characterized by a similar thickness, independent of the TO content. This phenomenon may result from the immiscibility of the systems due to the presence of hydrophobic substances of essential oil in the hydrophilic polymer matrix. The TO microdroplets introduced into the alginate film placed between alginate chains enlarged the distances between alginate macromolecules, which resulted in a looser network and, consequently, thicker films. The structure of the alginate film was probably spacious enough to hold a whole portion of the TO without an apparent increase in film thickness when the amount of TO in the films was higher. The increase in the thickness of polymeric films after the addition of essential oil was also observed by other researchers [50,51,54].

The moisture content in the studied films was determined, and the data were presented in Table 4. It is seen that the plasticized alginate film contained about 50% moisture because of the affinity of glycerol and alginate to absorb water molecules. At the same time, the films with TO had less moisture because hydrophobic components of TO prevented alginate and glycerol from absorbing water molecules. TO microdroplets were distributed in the alginate network, making the access of the hydroxyl groups in alginate and glycerol to bind water molecules complicated [52,54]. The moisture content in food packaging should be reduced due to the possible microorganisms’ growth in food in humid environments.

### 3.5. SEM Results

**Figure 2:**
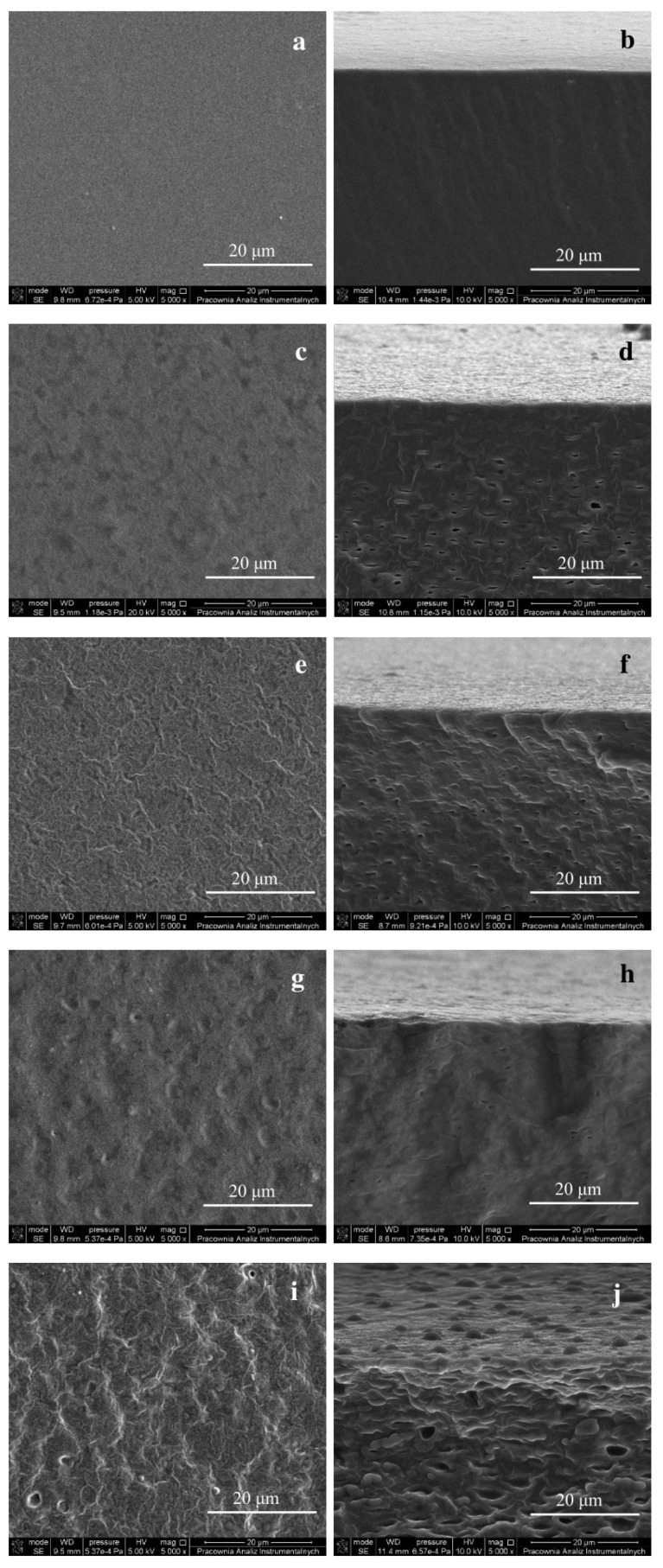
SEM images of the surface (left column) and fracture (right column) of the plasticized alginate films containing TO, viewed at a magnification of 5000×: Alg + G (**a**,**b**), Alg + G + TO 1% (**c**,**d**), Alg + G + TO 1.33% (**e**,**f**), Alg + G + TO 1.67% (**g**,**h**), and Alg + G + TO 2% (**i**,**j**).

Figure 2 shows microphotographs of the surfaces (left column) and fractures (right column) of the prepared films. It is seen that the plasticized alginate film exhibited a uniform smooth surface (Figure 2a), with no pores, hills, or valleys. When TO was added to the biopolymer, the films’ textures altered significantly, and the changes depended on the content of TO in the film. The surfaces of the film with TO became coarse with a fiber-like structure, which may result from the film components’ incompatibility due to the difference in their polarity. The film with the highest TO content (Figure 2i) had the roughest and most fibrous surface. The heterogeneous and coarse texture of biopolymeric films with essential oil was reported by other researchers [52,55,56].

The film fractures revealed rough and compact structures with about 1–5 μm diameter holes. It is worth recalling that the films were broken after freezing them in liquid nitrogen. This treatment affected the appearance of the cross-sections because the liquid compounds were removed during this procedure [46]. The visible holes were probably the places where these substances occurred. The holes were not observed in such amounts on the surfaces, which may indicate that volatile components of essential oil were trapped in the bulk of the films.

### 3.6. Thermal Analysis Results

**Figure 3:**
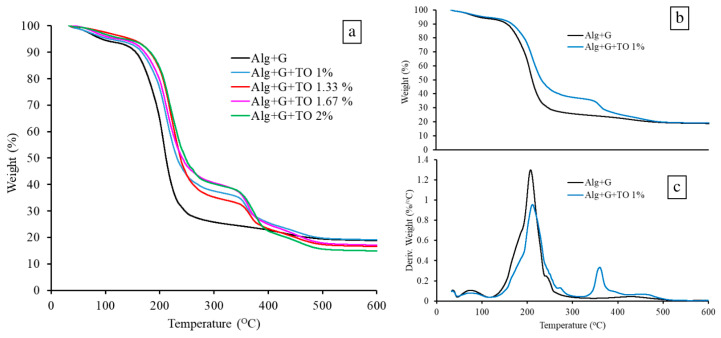
TG (**a**,**b**) and DTG (**c**) curves of the studied samples.

Thermogravimetric analysis was applied to test how the tansy essential oil influenced the thermal stability of the prepared samples. Figure 3 shows the studied films’ TG (a, b) and DTG (c) curves. It is seen that the thermal decomposition of all studied films started with a weight loss of less than 6% in the temperature 35–110 °C; the highest weight loss value was characteristic of the sodium alginate sample. This step was related to the release of water in the case of the alginate sample [57,58] and volatile compounds of essential oil in the case of alginate samples enriched in TO [59].

The main step of thermal decomposition occurred in the temperature range of 130–300 °C. In this step, the samples lost the most weight, 67% in the case of the sodium alginate sample and 55–60% in the samples with TO. The weight loss in this step was associated with alginate and glycerol decomposition [60] in the case of sodium alginate film and with the evaporation of essential oil compounds in the case of the films with TO [57,58]. The decomposition of alginate film without essential oil started at 179 °C. In contrast, degradation of the alginate samples with TO began at slightly higher temperatures (183 °C, 185 °C, 187 °C, and 188 °C for the samples with 1%, 1.33%, 1.67%, and 2% of TO, respectively), which indicated enhanced thermal stability of sodium alginate in the presence of TO due to the stronger interactions between components in the films. There are numerous publications in which the greater thermal stability of systems with essential oils have been described [59,60,61,62,63].

The next step in the temperature range of 310–410 °C, visible in TG and DTG curves of the alginate films with essential oil, could be related to the decomposition of the surfactant. Almasi A et al. conducted a thermogravimetric analysis of alginate film with essential oil and the surfactant (Tween 80) and the peak in the temperature range of 300–400 °C assigned to the decomposition of Tween 80 [59]. In this step, the weight loss was about 12–18%; the weight loss increased with the increasing content of TO and, at the same time, the surfactant.

The last step in the range of the highest temperatures, accompanied by weight loss of about 5–6%, was related to the decomposition of sodium alginate and the formation of Na_2_CO_3_ and the carbonized material [57]. The thermal decomposition of sodium alginate was not complete, as a residue of about 14–19% at 600 °C was observed in all samples. The amount of residue decreased when the content of TO in the film increased, suggesting that TO facilitated sodium alginate decomposition.

### 3.7. Antioxidant Capacity Results

The antioxidant properties of the films are favorable in terms of their usage as food packaging because such materials can prevent food from oxidation. Thus, introducing TO-containing active compounds to the alginate film aimed at acquiring films with antioxidant features. The plasticized sodium alginate film did not reveal any antioxidant properties. In contrast, the polymeric films with TO showed good AC (Table 4), which can be attributed to the presence of phenolic compounds, mainly phenolic acids [25,26,64]. Moreover, the AC increased with the increasing content of TO in the films owing to the higher content of phenolic compounds. These substances can efficiently react with free radicals, e.g., DPPH. This radical can undergo deactivation in two mechanisms: both single electron transfer (SET) and hydrogen atom transfer (HAT) reactions, resulting in the formation of an inactive reduced form of DPPH-H regardless of the method of deactivation [65].

### 3.8. Antibacterial Assay Results

**Figure 4:**
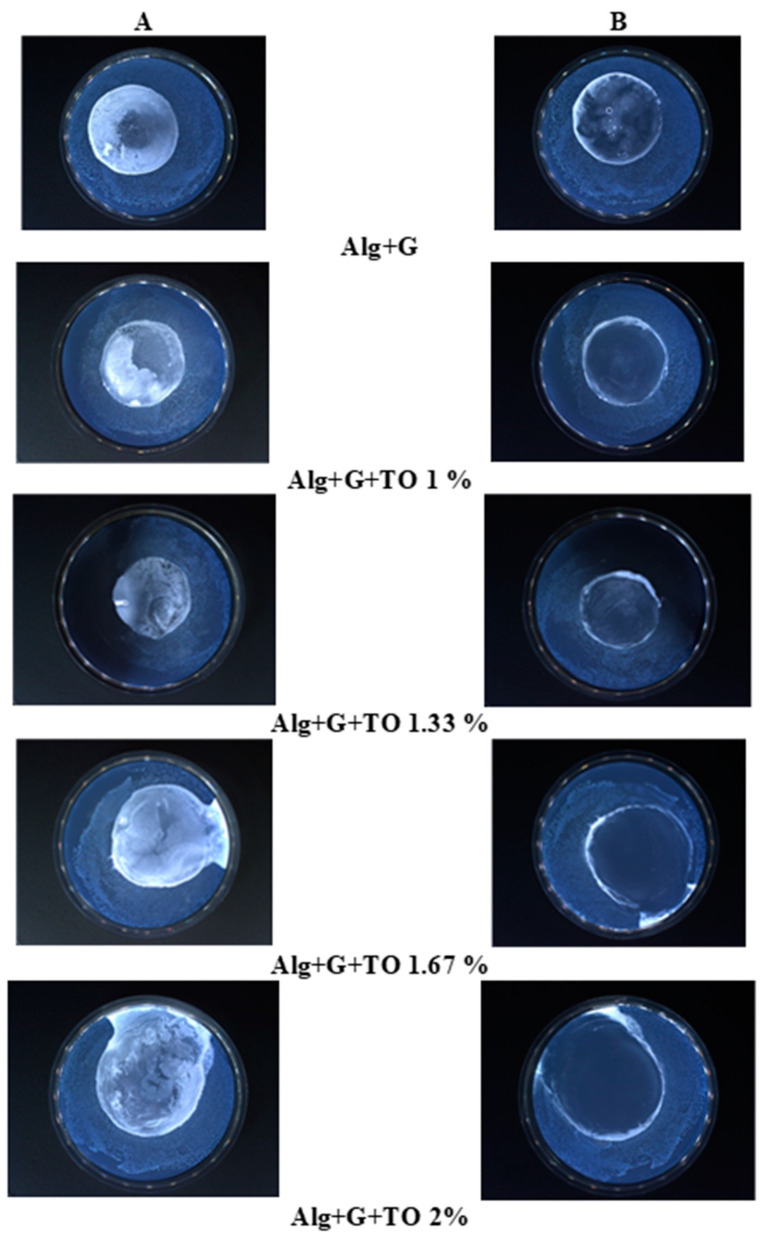
Growth of *S. aureus* in the area of contact of the sample with the agar ((**A**)—photos with the tested samples, (**B**)—photos after removing the tested samples).

The results of the tests for determining the antibacterial properties of the samples against *S. aureus*, *E. coli*, and *P. aeruginosa* bacteria strains are presented in Table 5 and Table 6, Figure 4 and Figures S1–S3. Table 5 shows the qualitative results for determining the antibacterial properties of the samples according to the ISO 20645:2006 standard [41]. The growth of bacteria on the nutrient solution under the working sample means whether the bacteria grew on the nutrient medium or not under the tested sample, “strong” means a lot of them, and “lack” means there was no bacteria growth. The assessment of the effect from the previous column according to the standard showed that the plasticized alginate film enabled the growth of bacteria strains. In contrast, the microorganisms’ growth in contact with the alginate films containing lower TO concentrations (1% and 1.33%) was limited, while the biopolymeric films with higher TO concentrations (1.67% and 2%) inhibited the bacteria proliferation.

Figure 4 shows the images of the sample actions against *S. aureus*. The images of the activity of *E. coli* and *P. aeruginosa* are presented in Figures S2 and S3. Figure S1 confirms the viability of the applied bacteria. The left column of Figure 2 presents photos of Petri dishes with inoculated agar medium on which the tested films were placed (white and turbid places in the middle of the Petri dishes). The right column presents the same Petri dishes after removing the tested films. The turbidity seen in the pictures in Figure 4 when the alginate film was taken off (Figure 4B: Alg + G) indicated the growth of bacteria on an agar medium under the film, while clear places after taking off the produced films enriched in TO pointed out the inhibition of bacteria growth under these films (Figure 4B: samples with 1.67% and 2% of TO). The active compounds in this essential oil are responsible for antibacterial properties.

Table 6 presents the quantitative results according to the ISO 22196 standard [42]. It is seen that the R value, meaning the number of viable bacterial cells on a surface, increased with an increasing amount of TO in the films [66]. Values above 2 for the samples with a higher content of tansy essential oil indicated good antibacterial properties of these materials. In contrast, R values below 2 were characteristic of the films with worse antibacterial activity. Moreover, all studied films exhibited similar antibacterial activity against Gram-negative bacteria (*E. coli*, *P. aeruginosa*) and Gram-positive bacteria (*S. aureus*). Previous research showed that tansy essential oil possessed antibacterial activity [25,26,67]. This research proved that TO retains its valuable properties when placed in alginate films. The presented test results, both in Table 5 and Table 6 and in Figure 4 and Figures S2 and S3, clearly proved that the samples with 1.67% and 2% TO showed antibacterial properties against *E. coli*, *S. aureus*, and *P. aeruginosa*, which are typical human pathogens causing nosocomial infections.

Essential oils contain a large number of varied components. The literature data showed that the overwhelming majority of the compounds in TO were oxygenated monoterpenes, which were responsible for antimicrobial properties [25,26,34]. In contrast, phenolic compounds to which phenolic acids and flavonoids belonged were responsible for antioxidant activity [25,68]. In the literature, the antioxidant activity of TO was assigned to phenolic acids such as caffeic and p-coumaric acid [68], caffeic, ferulic, and rosmarinic acid [25], neochlorogenic, 3,5-O-dicaffeoylquinic, and caffeoylquinic acid [26].

The antimicrobial activity of TO was attributed to terpenoids, which were the dominant group of compounds. Using ATR-FTIR spectroscopy and based on the literature, it was found that thujones, camphor, bornyl acetate, and 1,8-cineole could be the primary components of tansy essential oil used in this research. However, all the constituents in essential oil interact with each other, resulting in the overall activity of the oil. The prepared sodium alginate–based films with tansy essential oil effectively inhibited the growth of *E. coli*, *S. aureus*, and *P. aeruginosa*. These microorganisms are ubiquitous. *Staphylococcus aureus* is found on the skin, in the nose, and throat of humans and animals [69], *E. coli* lives in the intestines of humans and animals 69], while *Pseudomonas aeruginosa* usually inhabits water, soil, and vegetation and colonizes hospital equipment [70,71]. These bacteria can cause spoilage of food, and when they are transmitted to people with contaminated food, they cause diseases after consuming contaminated food. Tansy (*Tanacetum vulgare*) is a common plant containing thujones that are considered toxic. There are two natural isomers of thujones, (–)-α-thujone and (+)-β-thujone. Both thujones act as antimicrobial agents, but α-thujone was found to be more toxic than β-thujone [72]. However, not only thujones but also other components of the essential oils were responsible for the toxicity and antibacterial activity of the tansy essential oils and other oils acquired from plants rich in thujones, for instance, Sage (*Salvia officinalis* L.), absinthe wormwood (*Artemisia absinthium* L.), and Eastern arborvitae (*Thuja occidentalis* L.) [72]. Thujone-containing plants and their essential oil are allowed to be used in the food industry as a flavor additive [72,73].

Incorporating essential oils into biodegradable polymers forms active food packaging or coatings, which means that such packaging contains active substances that protect food from spoilage and prolong the shelf life of the products. The role of active compounds is to remove harmful agents, such as oxygen, moisture, and many others [74,75]. The components of essential oils possess antioxidant, antibacterial, and antifungal properties, which is an advantage in the food industry. The essential oils make the biopolymeric films active, while other properties of such films, such as mechanical, barrier, or opacity properties, should also be acceptable. These features of essential oils contributed to their use in packaging for seafood products to prevent spoilage [76], for bakery products against mold and yeast [77], and for meat products to protect from lipid and protein oxidation and microorganism growth [78].

## 4. Conclusions

Sodium alginate films with glycerol and tansy essential oil (*Tanacetum vulgare* L.) were prepared. Then, the physicochemical, thermal, antioxidant, and antibacterial properties of these films were examined. The microstructure of the films with TO was coarse and porous. The infrared spectra showed weak interactions between components in the films and a reduction in the amount of hydrogen bonding when TO was added. Moreover, the introduction of TO into the alginate films resulted in a decrease in moisture content, a growth in the film thickness, and a slight increase in the flexibility of the films. The films enriched with TO exhibited antioxidant properties. At the same time, films with higher concentrations of tansy essential oil showed good antibacterial properties against *E. coli*, *S. aureus*, and *P. aeruginosa*, making them suitable for food packaging. Moreover, these produced materials’ features make them attractive for cosmetic and medicinal applications.

## Figures and Tables

**Table 1:** Viscosities (mPa∙s) of alginate solutions with glycerol and tansy essential oil at different speeds (1.5–60 rpm); SD means standard deviation.

	Viscosity (mPa∙s) ± SD					
Sample	1.5 rpm	3 rpm	6 rpm	12 rpm	30 rpm	60 rpm
Alg + G	800 ± 23 ^a^	933 ± 5 ^b^	933 ± 8 ^d^	967 ± 16 ^a^	947 ± 12 ^c^	906 ± 5 ^b^
Alg + G + TO 1%	800 ± 11 ^a^	1022 ± 8 ^a^	1067 ± 9 ^b^	1111 ± 12 ^a^	1064 ± 33 ^a^	1033 ± 12 ^a^
Alg + G + TO 1.33%	800 ± 15 ^a^	1022 ± 13 ^a^	1133 ± 6 ^a^	1133 ± 12 ^a^	1098 ± 10 ^a^	1038 ± 10 ^a^
Alg + G + TO 1.67%	800 ± 13 ^a^	978 ± 11 ^b^	1067 ± 9 ^b^	1100 ± 7 ^a^	1080 ± 11 ^a^	1029 ± 4 ^a^
Alg + G + TO 2%	800 ± 17 ^a^	933 ± 16 ^b^	1000 ± 7 ^c^	1011 ± 23 ^a^	1004 ± 9 ^b^	953 ± 7 ^c^

Different superscripts (a–d) within the same column indicate significant difference between the compared values (*p* < 0.05).

**Table 2:** The absorption band maxima and their assignment to the vibrational modes for sodium alginate film plasticized with glycerol [48,49].

Wavenumber (cm^−1^)	Vibrational Mode
3266	O–H stretching
2932	C–H asymmetric stretching
2881	C–H symmetric stretching
1603	COO^–^ asymmetric stretching
1407	COO^–^ symmetric stretching
1095	C–O–C asymmetric stretching in the glycosidic bond
1026	C–O–C asymmetric stretching
995	C–O asymmetric stretching (in C–O–H)
924	O–H deformation (in glycerol)
851	C–O symmetric stretching (in glycerol)
816	C–O–C symmetric stretching

**Table 3:** Young’s modulus (E, MPa), stress at break (σ, MPa), strain at break (ε, %) of the studied samples; SD means standard deviation.

Sample	E(MPa) ± SD	σ (MPa) ± SD	ε (%) ± SD
Alg + G	10.48 ± 1.49 ^a^	7.10 ± 1.47 ^a^	52.29 ± 1.78 ^ab^
Alg + G + TO 1%	7.83 ± 1.39 ^b^	6.88 ± 0.97 ^ab^	61.81 ± 4.88 ^a^
Alg + G + TO 1.33%	9.13 ± 0.67 ^a^	6.02 ± 0.44 ^ab^	50.16 ± 3.58 ^b^
Alg + G + TO 1.67%	7.27 ± 0.57 ^b^	5.31 ± 0.46 ^b^	53.48 ± 4.07 ^ab^
Alg + G + TO 2%	6.89 ± 0.64 ^b^	5.22 ± 0.63 ^b^	56.19 ± 4.82 ^ab^

Different superscripts (a,b) within the same column indicate significant difference between the compared values (*p* < 0.05).

**Table 4:** Thickness (mm), moisture content (MC, %), and antioxidant activity (AC, μmol Trolox/100 g) of the studied samples; SD means standard deviation.

Sample	Thickness (mm) ± SD	MC (%)± SD	AC ± SD
Alg + G	0.108 ± 0.005 ^a^	50.32 ± 1.38 ^a^	0 ^a^
Alg + G + TO 1%	0.145 ± 0.007 ^b^	35.50 ± 0.08 ^b^	136.69 ± 4.98 ^b^
Alg + G + TO 1.33%	0.143 ± 0.010 ^b^	29.40 ± 1.67 ^c^	138.96 ± 6.46 ^b^
Alg + G + TO 1.67%	0.146 ± 0.012 ^b^	19.48 ± 0.98 ^d^	292.91 ± 7.00 ^c^
Alg + G + TO 2%	0.148 ± 0.013 ^b^	14.32 ± 0.12 ^e^	280.08 ± 8.39 ^c^

Different superscripts (a–e) within the same column indicate significant difference between the compared values (*p* < 0.05).

**Table 5:** The results of antibacterial activity against *E. coli*, *S. aureus*, and *P. aeruginosa*.

Sample	Growth of Bacteria on the Nutrient Solution under the Working Sample	Rating of Antibacterial Effect
*E. coli*		
Alg + G	strong	insufficient effect
Alg + G + TO 1%	average	limited efficiency
Alg + G + TO 1.33%	average	limited efficiency
Alg + G + TO 1.67%	lack	good effect
Alg + G + TO 2%	lack	good effect
*S. aureus*		
Alg + G	strong	insufficient effect
Alg + G + TO 1%	average	limited efficiency
Alg + G + TO 1.33%	average	limited efficiency
Alg + G + TO 1.67%	lack	good effect
Alg + G + TO 2%	lack	good effect
*P. aeruginosa*		
Alg + G	strong	insufficient effect
Alg + G + TO 1%	average	limited efficiency
Alg + G + TO 1.33%	average	limited efficiency
Alg + G + TO 1.67%	lack	good effect
Alg + G + TO 2%	lack	good effect

**Table 6:** Antibacterial activity of the studied samples against human pathogens.

Sample	Bacteria Strain	R	% Reduction
Alg + G	*E. coli*	0.32	<90.0
Alg + G + TO 1%		1.04	>90.0–99.0
Alg + G + TO 1.33%		1.26	>90.0–99.0
Alg + G + TO 1.67%		2.16	>99.9
Alg + G + TO 2%		2.30	>99.9
Alg + G	*S. aureus*	0.44	<90.0
Alg + G + TO 1%		1.12	>90.0–99.0
Alg + G + TO 1.33%		1.42	>90.0–99.0
Alg + G + TO 1.67%		2.02	>99.9
Alg + G + TO 2%		2.32	>99.9
Alg + G	*P. aeruginosa*	0.50	<90.0
Alg + G + TO 1%		1.40	>90.0–99.0
Alg + G + TO 1.33%		1.60	>90.0–99.0
Alg + G + TO 1.67%		2.10	>99.9
Alg + G + TO 2%		2.20	>99.9

## Data Availability

Data are available from the authors upon request.

## Supplementary Materials

The following supporting information can be downloaded at: https://www.mdpi.com/article/10.3390/polym15020260/s1. Figure S1. Bacterial growth (*E. coli*, *S. aureus*, *P. aeruginosa)*—samples verifying the viability of bacteria; Figure S2. Growth of *E. coli* in the area of contact of the sample with the agar (A-photos with a sample, B-photos after removing the tested samples); Figure S3. Growth of *P. aeruginosa* bacteria in the area of contact of the sample with the agar (A-photos with a sample, B-photos after removing the tested samples).
