# Supplementary material for: Physicochemical and Antibacterial Properties of Alginate Films Containing Tansy (Tanacetum vulgare L.) Essential Oil
Source: Polymers (Basel). 2023 Jan 4;15(2):260. doi: 10.3390/polym15020260 (PMC9866307; doi:10.3390/polym15020260)
Supplement: Supplementary file 1 [file polymers-15-00260-s001.zip › polymers-2096915-supplementary.pdf]

Supplementary Materials

Physicochemical and Antibacterial Properties of Alginate Films Containing Tansy (*Tanacetum vulgare* L.) Essential Oil

Jolanta Kowalonek [1,*], Natalia Stachowiak [1], Kinga Bolczak [1] and Agnieszka Richert [2]

[1] Faculty of Chemistry, Nicolaus Copernicus University in Torun, Gagarina St. 7, 87-100 Torun, Poland
[2] Department of Genetics, Faculty of Biological and Veterinary Sciences, Nicolaus Copernicus University in Torun, Lwowska St. 1, 87-100 Torun, Poland
* Correspondence: jolak@umk.pl

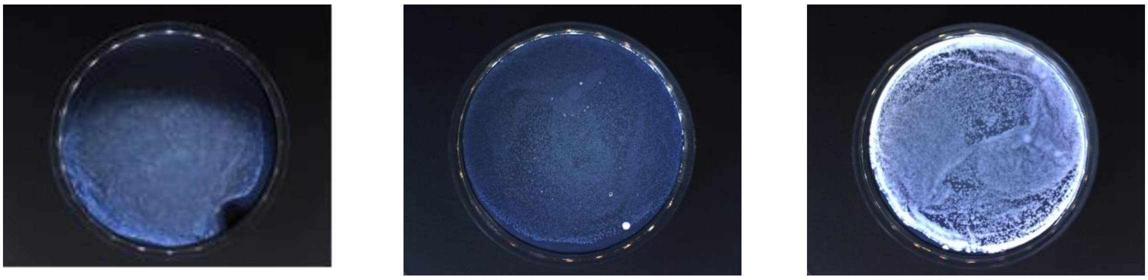

E. coli *S. aureus* *P. aeruginosa*

Figure S1. Bacterial growth (*E. coli*, *S. aureus*, *P. aeruginosa)* - samples verifying the viability of bacteria.

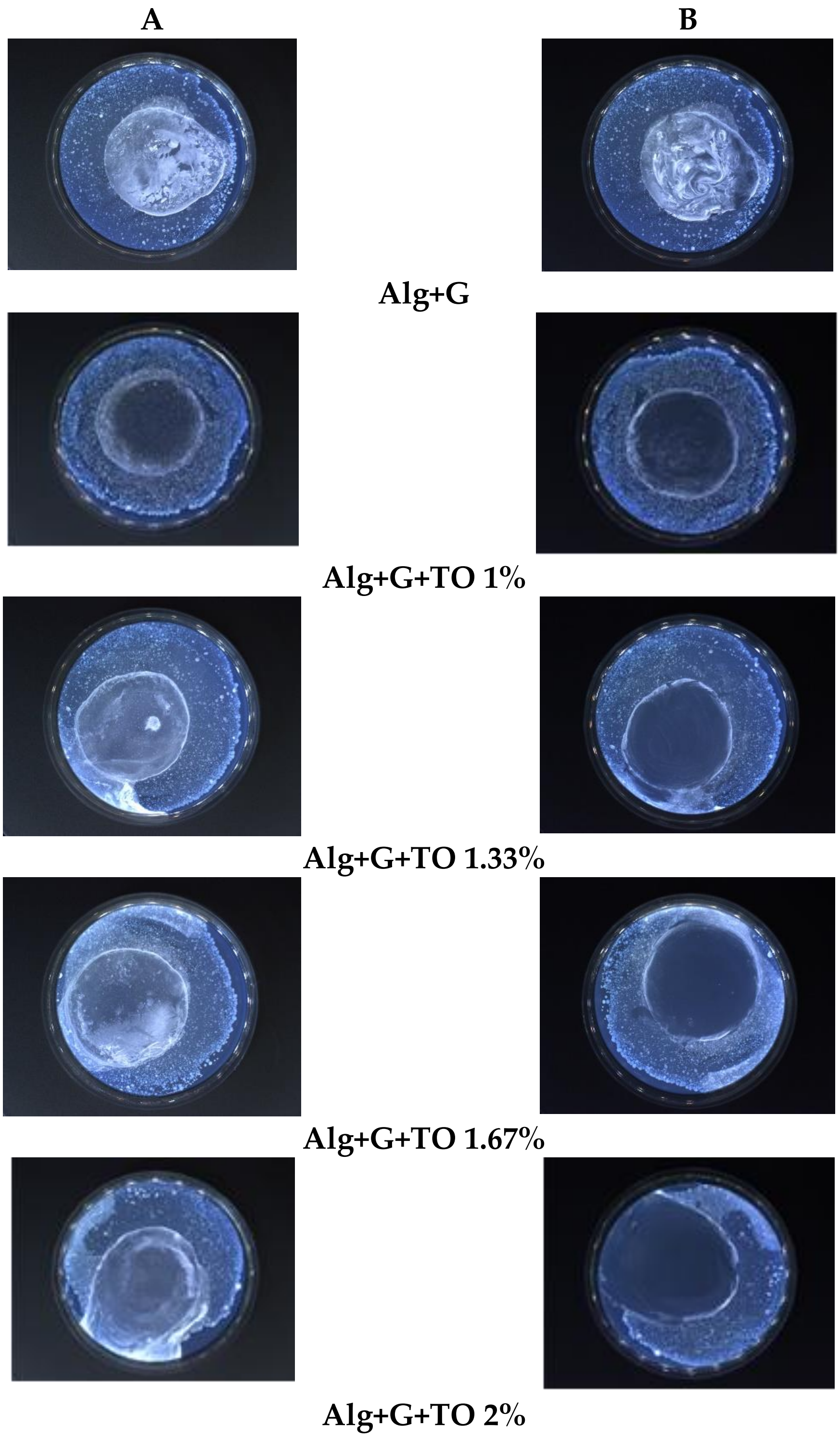

Figure S2. Growth of *E. coli* in the area of contact of the sample with the agar (A-photos with a sample, B- photos after removing the tested samples).

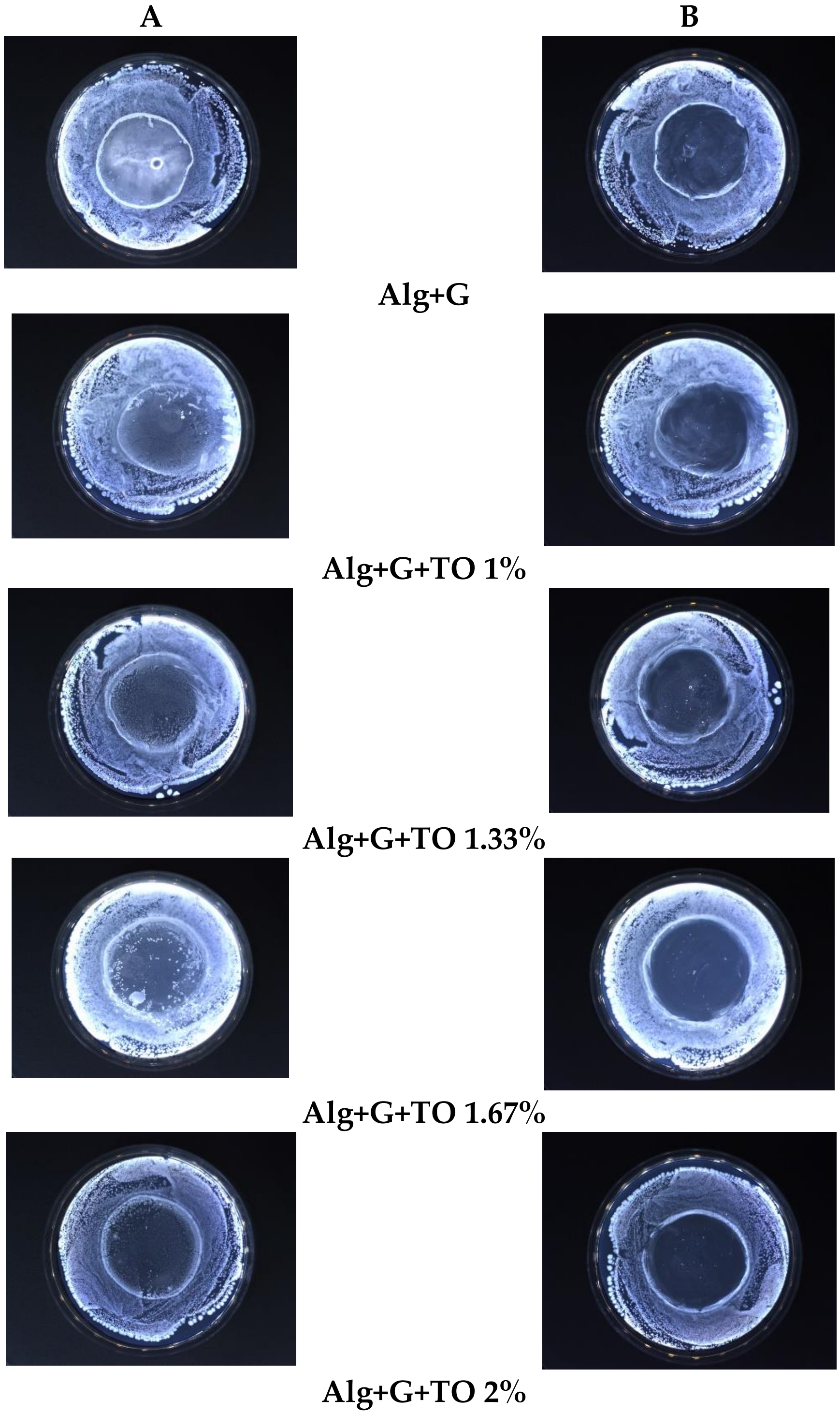

Figure S3. Growth of *P. aeruginosa* bacteria in the area of contact of the sample with the agar (A- photos with a sample, B- photos after removing the tested samples).
